# Association between Hematological Parameters and Severity of Covid-19 Infections

**DOI:** 10.12688/f1000research.148734.1

**Published:** 2024-05-20

**Authors:** Gokul Krishnan, Shubhada Karanth, Sudha Vidyasagar, Archit Aggarwal, Anurupa Udupi, Suresh Karanth, Shivashankara Kaniyoor Nagri

**Affiliations:** 1 Department of General Medicine, Kasturba Medical College Manipal, Manipal Academy of Higher Education, Manipal, Karnataka, 576104, India; 2 General Medicine, The Voluntary Health Services Hospital, Chennai, Tamil Nadu, India; 3 Hematology and hematooncology, A J Institute of Medical Sciences and Research center, Mangalore, Karnataka, India

**Keywords:** COVID-19; Droplet spread; Inflammation; Hematological; Pandemic; SARS-CoV-2

## Abstract

**Background:**

This study aimed to determine the relationships between hematological parameters- hemoglobin, Total Leucocyte Counts (TLC), platelet counts, Absolute Neutrophil Counts (ANC), Absolute Lymphocyte Counts (ALC), Neutrophil Lymphocyte Ratio (NLR), Systemic Immune Inflammatory Index (SII), Neutrophil Monocyte Ratio (NMR), Platelet Lymphocyte Ratio (PLR) and the severity of COVID 19 infections and their use in predicting severity of COVID-19 infections.

**Methods and Material:**

This was a prospective, observational, single-center study of 573 symptomatic adult inpatients of COVID 19 admitted to our tertiary care center.

**Statistical analysis used:**

The above-mentioned hematological parameter levels were noted and compared between the two categories of COVID-19 infection, namely non-severe and severe COVID-19 using logistic regression methods. Their cut-off values were detected using the ROC curve.

**Results:**

The median TLC, ANC, NLR, SII, NMR, PLR were notably higher in patients with severe COVID-19 than in those with non-severe COVID-19. Logistic regression analysis showed that NMR (OR=1.029, p=0.006) and ALC (OR=0.999, p=0.002) were statistically significant independent predictors of COVID-19 severity

**Conclusions:**

The hematological parameters mentioned, can be used for predicting severe COVID-19 infections at admission. ALC and NMR levels could be used as hematological markers to predict severity of COVID-19 in adult patients with their cut off values being < 1105 cells/cubic millimeter and > 10.434 respectively.

## Introduction

In early December 2019, the world saw the beginning of an epidemic of the new coronavirus disease 2019 (COVID-19), due to the newfound virus, severe acute respiratory syndrome coronavirus 2 (SARS-CoV-2). COVID-19 later evolved into a pandemic that affected billions of people worldwide. Measures taken for social distancing and the impact of the disease on socio-economic status, morbidity, and mortality have proven to be a major burden on millions of people all over the world. ^1^

India saw its first case in Kerala in late January 2020. ^2^ India with a total of 430422 active cases as of 16/07//2021 as per the Ministry of Health and Family Welfare, Government of India website. ^3^ Mortality rates due to the disease have varied from 3% in China to 145% in some cities in Mexico. ^4^^,^^5^ India has a mortality rate of 1.33% as of 16/07/2021. ^3^ A resurgence has been noted with JN1 as the new variant with high immune evasion properties. ^6^ Thus, simple tools for triaging and prognosticating COVID-19 are needed. Studies on hematological parameters of COVID-19 in our country are scarce. This study is a modest attempt to study the association of basic hematological parameters such as total leukocyte count, neutrophil-to-monocyte ratio (NMR), neutrophil to lymphocyte ratio (NLR), Systemic Inflammatory Index (SII), and platelet-to-lymphocyte ratio (PLR), which can be easily availed even in rural setups, with the severity of COVID-19, which might aid in the evaluation of patients and in detecting the progression to severe disease at the time of admission. This may even help in the judicious triaging of patients at the time of admission.

## Methods

A prospective observational study was conducted by clinical examinations and lab investigation in the Department of Internal Medicine, at a tertiary care hospital, in Karnataka, India for a period of three months from 03/12/2020 to 25/03/2021.

The study was approved by our Institutional Review Board (IRB) known as Kasturba Medical College and Kasturba Hospital Institutional Ethics Committee (reference number: 740/2020). It was approved on 3 ^rd^ December, 2020 for a period of three months till 31 ^st^ March 2021. This study was conducted in accordance with the principles of the Declaration of Helsinki. Written informed consent was waved and telephonic verbal consent was taken citing restrictions during COVID-19. The same was approved by the Institutional Ethics Committee. The data collected were kept confidential.

### Inclusion and exclusion criteria

All symptomatic adult COVID-19 patients who were admitted to our tertiary care facility in Karnataka, India, and whose diagnoses were made by nasopharyngeal swab RT-PCR were included in this prospective study. The inclusion criteria were hospital admissions of patients who tested positive for COVID-19 by RT-PCR and were at least 18 years of age.

Patients with the following conditions were not included in the study: patients with known cases of sepsis or other associated infections at the time of COVID-19 diagnosis; patients with diagnosed cases of cytopenia, including autoimmune cytopenia, Evans disease, and ITP; patients with diagnosed cases of congenital or acquired bone marrow failure; patients with diagnosed cases of primary haematological malignancies or malignancies involving bone marrow; and patients who were discharged against medical advice.

A total of 622 patients were included in this study. Even so, nine patients declined to take part in the study, four individuals revoked their consent while receiving treatment at the hospital, and 27 people were excluded from the study altogether. Nine patients were discharged against medical advice.

Final sample size in this study was 573.

The study collected demographic information including age and sex; clinical data including symptoms; a history of comorbidities including diabetes mellitus, essential hypertension, chronic kidney disease, diagnosed malignancies, and ischemic heart disease; as well as the results of general and systemic examinations. A complete blood profile, arterial blood gas analysis (ABG), liver and renal function tests, an electrocardiogram, and a chest X-ray were among the laboratory investigations performed. Further examinations were conducted in adherence to the protocol prescribed.

Normal hemoglobin levels were taken as - 12-15 g/dL, total leukocyte counts as those between 4000-10000 cells per cubic millimeter, neutrophil counts as 2000-8000 cells per cubic millimeter, lymphocyte counts as 720-4400 cells per cubic millimeter, and platelet counts as 150000-400000 cells per cubic millimeter.

All COVID-19 patients in the study were classified as mild, moderate, severe and critical COVID-19 at the time of admission. This was done using the national guidelines provided by the Indian Council of Medical Research, which was similar to the WHO guidelines. ^7^ Those with mild symptoms maintaining oxygen saturation >90% on room air were considered to be in the non-severe category. The patients with moderate, severe and critical symptoms who required oxygen supports and/or required intensive care unit admission were classified as severe.

The patients were periodically reviewed during their in-hospital stay, and the highest degree of disease severity was considered when classifying the cohorts.

Nasopharyngeal throat swabs for COVID-19 RTPCR were obtained from all patients enrolled in the study in the hospital virology laboratory using the TRUPCR RT qPCR kit. It is a reagent system based on real-time PCR technology for the detection of diseases.

The protocol established by the ICMR (Indian Council of Medical Research) were used for performing RT-PCR assays. This was in accordance with the WHO guidelines.

EDTA whole-blood samples were used for complete blood counts.

The Systemic Immune Inflammatory Index was calculated using the formula - Neutophil counts/Lymphocyte counts and platelet counts.

### Statistical analysis

An analysis was conducted on all categorical variables, including underlying comorbidities and sex, and the results were presented as frequencies and percentages. When applicable, continuous variables exhibiting skewed distributions, such as TLC and ANC, were denoted using the median along with the interquartile range (Q1-Q3). Conversely, variables following normal distributions were depicted using the mean ± standard deviation. Using the Mann-Whitney U test, the difference between the medians of the non-severe and severe COVID-19 categories was analysed. By employing logistic regression analysis, the optimal parameters for predicting the severity of Covid-19 were identified. The specificity and sensitivity of the test were ascertained, as well as the cut-off values for different parameters pertaining to the non-severe and severe COVID-19 groups, utilising a receiver operating characteristic curve. The software SPSS 23.0 (IBM SPSS statistics, USA) was utilised to analyse the data.

## Results

The demographic and baseline characteristics of the patients are shown in Tables 1 and 2. Among the 573 COVID-19 patients in our study, the majority were males. In the severe category, many patients in the ICU required mechanical ventilation and non-invasive ventilation. The rest were administered oxygen supplementation via a Venturi mask, non-rebreathing masks (NRBM) and nasal prongs. Comorbidities were observed in a large number of patients. Overlap of more than one chronic illness was unavoidable in some patients.

**Table 1.** Demographic and baseline characteristics of patients diagnosed with COVID-19 infection.

Characteristics	Distribution
**Demographics and comorbid conditions**	
Age (years) ^†^	55.22±15.84
Male [ *n* (%)]	353 (61.6)
Female [ *n* (%)]	220 (38.4)
Comorbidities [ *n* (%)]	492 (85.9)
Type 2 diabetes mellitus [ *n* (%)]	245 (42.8)
Essential hypertension [ *n* (%)]	223 (39)
Ischemic heart disease [ *n* (%)]	118 (20.6)
**Oxygen supports**	
Mechanical ventilation [n (%)]	96 (16.75)
Non- invasive ventilation [n (%)]	49 (8.55)
Others [n (%)]	157 (27.40)
**Laboratory features**	
Hemoglobin (mg/dL) ^†^	12.27 ± 2.14
Total white blood counts (cells/μL) ^‡^	7800.00 (5625-10900)
Absolute Neutrophil counts (cells/μL) ^‡^	5460.00 (3650-8490)
SII Index (Number × 10 ^10^/L) ^‡^	106.8110 (55.62-240.50)

Data are expressed in

^†^ (Mean ± Standard deviation) or in

^‡^ Median (Q1-Q3).

**Table 2.** Demographic and baseline characteristics of non-severe and severe COVID-19 patients.

		Non-Severe [ *n* (%)] ( *n:*271)	Severe [ *n* (%)] ( *n*:302)	P value
Age (years)	≤30	38 (14.02)	10 (3.31)	<0.001 *
	31-40	49 (18.08)	29 (9.60)	
	41-50	38 (14.02)	39 (12.91)	
	51-60	62 (22.87)	77 (25.49)	
	61-70	50 (18.45)	80 (26.49)	
	≥70	34 (12.54)	67 (22.18)	
Gender	Male	138 (50.92)	215 (71.19)	<0.001 *
	Female	133 (49.07)	87 (28.80)	
Comorbidities		202 (41.1)	290 (58.9)	
Type 2 Diabetes Mellitus ^†^		69 (28.2)	176 (71.8)	<0.001 *
Essential Hypertension ^†^		74 (33.2)	149 (66.8)	<0.001 *
Ischemic heart disease ^†^		40 (33.9)	78 (66.1)	0.001 *

* Statistically significant.

^†^ Parameters subjected to logistic regression analysis.

The median values of hematological parameters such as hemoglobin, total leukocyte counts, ANC, ALC, platelets, NLR, SII, NMR, and PLR were compared, and the statistically significant values are shown in Table 3. The hematological parameters were also compared between the survivors and non-survivors of the severe category, which are depicted in the same table. The mean hemoglobin values of the non-severe and severe COVID-19 categories were compared but were found to be statistically insignificant.

**Table 3.** Comparison of mean/median values of various hematological parameters in non-severe vs severe COVID 19 infections.

Hematological parameters	Non-severe ( *n*:271)	Severe (n:302)	P value	Severe		P value
				Survivors	Non Survivors	
Hemoglobin (g/dL)	12.36±2.08 ^†^	12.15±2.24 ^†^	0.223	12.17±2.20 ^†^	12.11±2.38) ^†^	0.211
Total Leucocyte Counts (cells/μL)	7200.00 (5500-10300) ^‡^	8300.00 (5700-12300) ^‡^	0.004 *	8000 (5425-11275) ^‡^	10250 (6925-14900) ^‡^	0.03 *
ANC (cells/μL)	4621.50 (3365-7067.5) ^‡^	6420.00 (4120-10095) ^‡^	<0.001 *	5765 (3730-9317) ^‡^	8775 (5097-12005) ^‡^	0.002 *
ALC (cells/μL)	1470.00 (970-2015) ^‡^	900.00 (595-1290) ^‡^	<0.001 *	915 (672-1290) ^‡^	830 (432-1307) ^‡^	0.097
Platelet Counts (cells/μL)	232000.00 (177000-291000) ^‡^	228000.00 (165000-315500) ^‡^	0.773	23600 (168250-312250) ^‡^	216500 (151750-331500) ^‡^	0.399
NLR	3.27 (2.02-5.75) ^‡^	7.54 (3.7-14.17) ^‡^	<0.001 *	7.31 (3.50-12.42) ^‡^	10.5 (4.74-19.77) ^‡^	0.005 *
SII Index (Number × 10 ^10^/L)	76.43 (42.02-142.61) ^‡^	158.40 (80.73-369.98) ^‡^	<0.001 *	146 (77.26-339.03) ^‡^	249.89 (83.02-534.96) ^‡^	0.02 *
NMR	8.00 (5.63-11.33) ^‡^	11.42 (7.7-18.4) ^‡^	<0.001 *	11 (7.43-17.60) ^‡^	14.46 (8.30-23.43) ^‡^	0.009 *
PLR	154.24 (108.15-240.43) ^‡^	244.31 (159.13-424.44) ^‡^	<0.001 *	237.74 (164.87-401.56) ^‡^	262.19 (146.51-561.17) ^‡^	0.440

Data are expressed in

^†^ (Mean ± Standard deviation) or in

^‡^ Median (Q1-Q3).

* Statistically significant.

ANC, absolute neutrophil count; ALC, absolute lymphocyte count; NLR, neutrophil-lymphocyte ratio; SII, systemic immune-inflammatory index; NMR, neutrophil-monocyte ratio; PLR, platelet-lymphocyte ratio.

COVID-19 patients were categorized into two groups. The non-severe and severe categories consisted of 271 and 302 patients, respectively. Statistically significant differences were only observed in patient age, sex, and associated comorbidities. Associated comorbidities were further analyzed using multivariate logistic regression, is shown in Table 4.

**Table 4.** Logistic Regression analysis for hematological parameters between Non-Severe and Severe COVID-19.

Parameters	OR (95% C.I)	P value
Total Leucocyte Counts (cells/μL)	0.999 (0.999–1.000)	0.708
ANC (cells/μL)	1.000136 (1.000070–1.000202)	0.214
ALC (cells/μL)	0.999 (0.999–1.000)	0.002 *
NLR	1.021 (0.985–1.060)	0.258
SII Index (Number × 10 ^10^/L)	1.001 (0.999–1.002)	0.354
NMR	1.029 (1.008–1.051)	0.006 *
PLR	0.999 (0.999–1.000)	0.657
Type 2 Diabetes Mellitus	3.325 (2.286–4.836)	<0.001 *
Essential Hypertension	1.689 (1.153–2.476)	0.007 *
Ischemic Heart Disease	1.513 (0.961–2.381)	0.073

* Statistically significant.

ANC, absolute neutrophil count; ALC, absolute lymphocyte count; NLR, neutrophil-lymphocyte ratio; SII, systemic immune-inflammatory index; NMR, neutrophil-monocyte ratio; PLR, platelet-lymphocyte ratio.

Comorbidities, such as type 2 diabetes mellitus, essential hypertension, ischemic heart disease, and the abovementioned hematological parameters, which were statistically significant, were further subjected to logistic regression ( Table 4). The hematological parameters between the survivors and non-survivors in the severe category of statistical significance were also subjected to logistic regression, but none of them were statistically significant. This analysis showed that type 2 diabetes mellitus, essential hypertension, NMR, and ALC were statistically significant independent predictors of COVID-19 severity while ischemic heart disease, as an independent predictor, had borderline significance.

**Figure 1.**
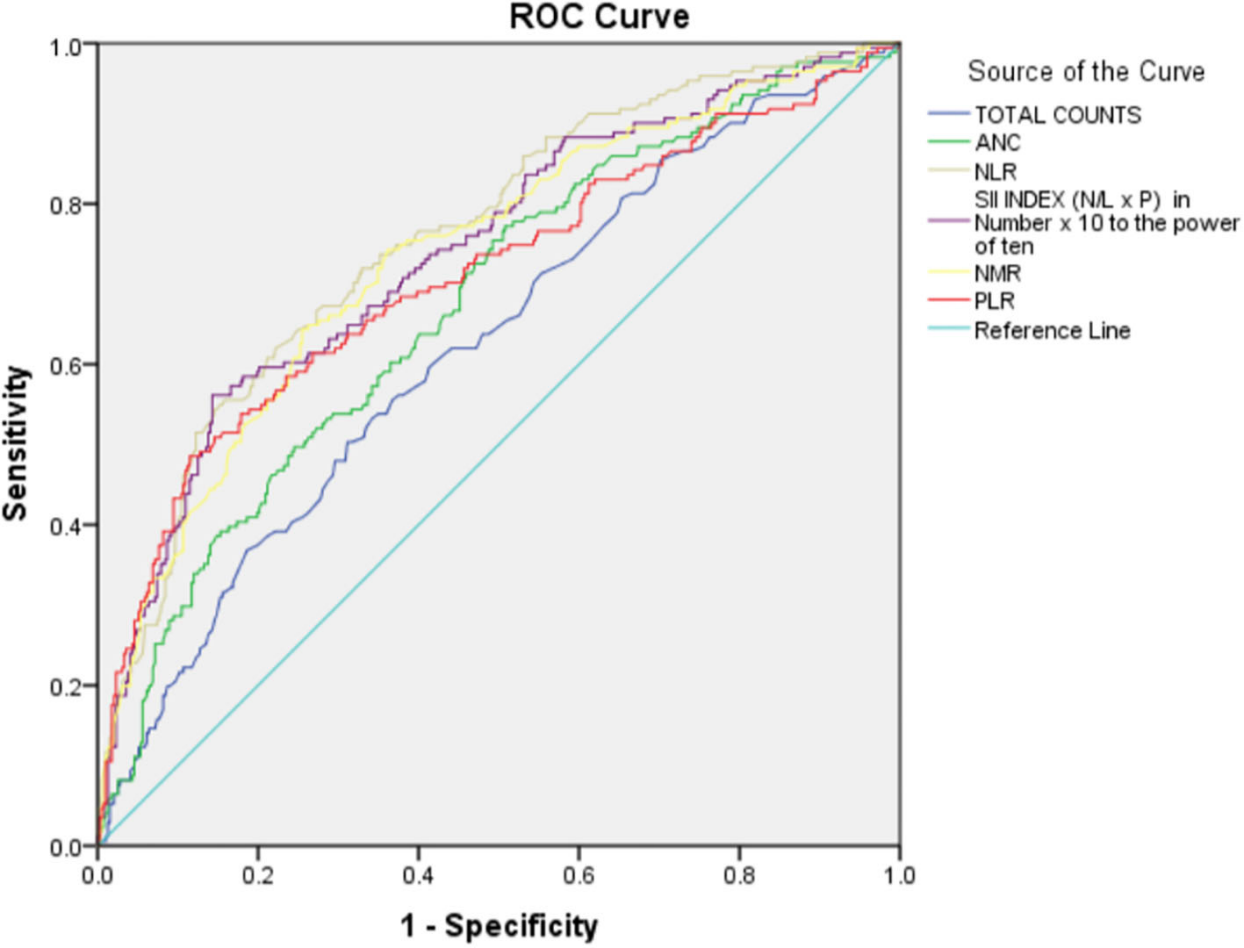
Receiver Operating Characteristic (ROC) curve for hematological parameters (a and b) with COVID-19 severity.

As there was a statistically significant difference between non-severe and severe categories in the above-mentioned hematological parameters, they were further studied using Receiver Operating Characteristic (ROC) curve analyses. Regarding the observations made by ROC analyses, the following information concerning patients with COVID-19 diagnosis severity was found ( Table 5 and Figure 1).

**Table 5.** Recommended cut-off values for significant markers in the prediction of severity of COVID-19 infections in patients.

Hematological parameters	AUC	95% C.I. (Q1-Q3)	p value	Cut off	Sensitivity (%)	Specificity (%)
Total Leucocyte Counts (cells/μL)	0.624	0.574-0.674	<0.001 *	>7850	62	56
ANC (cells/μL)	0.674	0.626-0.722	<0.001 *	>5745	64	60
ALC (cells/μL)	0.711	0.664-0.758	<0.001 *	<1105	70	60
NLR	0.759	0.716-0.803	<0.001 *	>5.665	72	68
SII Index (Number × 10 ^10^/L)	0.740	0.694-0.786	<0.001 *	>120.991	68	64
NMR	0.735	0.690-0.781	<0.001 *	>10.434	70	67
PLR	0.710	0.660-0.759	0.001 *	>198.71	69	61

* Statistically significant values.

ANC, absolute neutrophil count; ALC, absolute lymphocyte count; NLR, neutrophil-lymphocyte ratio; SII, systemic immune-inflammatory index; NMR, neutrophil-monocyte ratio; PLR, platelet-lymphocyte ratio.

## Discussion

This study was a prospective observational study that determined the mean age of the patients to be 55.82 ± 15.84 years. This conclusion aligns with earlier studies conducted in China and the USA. ^8^^–^^10^ However, the mean age in this study was slightly higher than that reported in an Indian cohort study conducted by Nitesh Gupta and colleagues in New Delhi. ^11^ The prevalence of the condition was higher in men (61.6%) compared to women, which is consistent with the results reported by Fei Zhou and his colleagues in China. ^9^ Richardson et al. conducted a study in the United States using a case series of 5700 patients. Their findings revealed that the death rate for males was consistently greater than that of females in every 10-year age interval beyond 20 years. ^10^ A total of 85.9% of patients had comorbidities. Our study found a considerable prevalence of type 2 diabetes mellitus and essential hypertension, particularly in the severe category. Our study demonstrated a marginal level of significance indicating that ischemic heart disease can be used as a predictor of the severity of COVID-19. Nevertheless, research conducted in China and the USA revealed that patients with COVID-19 were more likely to have essential hypertension as a pre-existing condition, with additional comorbidities such as type 2 diabetes mellitus and coronary artery disease following suit. ^8^^–^^10^

The results of our investigation indicated a little elevation in the overall number of white blood cells and neutrophils in those with severe illness. This finding aligns with the retrospective study undertaken by Lin et al. and Rami M. Elshazli et al. ^12^^,^^13^ A study conducted by Agrawal et al. in Rajasthan, India, found similar results in 102 patients who had both symptomatic and asymptomatic cases of COVID. ^14^ However, Guan and colleagues observed a higher incidence of leukopenia (33%) in the severe group compared to the non-severe group in their analysis of 1099 COVID-19 patients in China. ^8^ Neutrophils, monocytes, and lymphocytes, which are diverse components of white blood cell counts, have distinct functions in the immune response within our body. Neutrophils, the predominant kind of white blood cells in the peripheral blood of humans, are now recognised as having a significant impact on safeguarding the respiratory epithelium by promoting the production of pro-inflammatory cytokines in the immediate area. ^15^ Nevertheless, it appears to have both positive and negative consequences, since excessive activation of neutrophils can lead to heightened inflammation, increased tissue damage, and hence exacerbate the disease. ^15^^,^^16^

The results of our investigation revealed lymphocytopenia in the severe group, accompanied by a significantly elevated median NLR (neutrophil-to-lymphocyte ratio) and PLR (platelet-to-lymphocyte ratio). W. Guan and his colleagues also observed this significant lymphocytopenia. ^8^ Abhishek Agrawal and his colleagues in India observed a decrease in the average number of lymphocytes (20.58±0.87 vs 28.60±8.65), an increase in the average NLR (6.17±6.11 vs. 2.67±1.32), and an increase in the average PLR (146.29±88.90 vs. 115.47±47.88) among patients in the moderate to severely ill category, compared to asymptomatic and mildly symptomatic patients. ^14^ The study conducted by Sha-Lin et al. in China demonstrated that an increased NLR (neutrophil-to-lymphocyte ratio) is a standalone predictive indicator for the severity of COVID-19. ^12^ Lymphocytes are a specific type of white blood cells (WBCs) that play a crucial role in triggering adaptive immunity and the establishment of ‘self-tolerance’. ^17^^,^^18^

The cytokine storm typically occurs 1-2 weeks after the infection starts, when the virus moves through the circulation and primarily targets tissues with a high concentration of ACE2, the receptor for SARS-CoV-2. During this time, there is a noticeable decrease in lymphocyte count, known as lymphopenia. ^19^ Studies have demonstrated that lymphocytes in the blood express a limited quantity of ACE2 receptors on their surface. This is believed to be the reason of lymphopenia in COVID-19. ^20^ Monocytes are a distinct subset of white blood cells (WBCs) found in the human body. They can be categorised into three subtypes: classical, intermediate, and non-classical. ^21^ Recent investigations have indicated a decrease in intermediate and non-classical monocytes after SARS-CoV-2 infections. ^22^ These monocytes have the capacity to transform into activated macrophages and exhibit both anti-inflammatory and pro-inflammatory functions due to their capability to generate certain cytokines. Therefore, reducing them may disturb this equilibrium and lead to an elevation in the immunological response. ^23^

Neutrophils, lymphocytes, and monocytes have diverse functions in the immune response during various situations, such as COVID-19 infections. Therefore, it can be inferred that an elevated NMR could indicate a disturbance in the balance of the immune system cells, perhaps indicating the severity of the disease with considerable morbidity.

The Systemic Immune Inflammatory Index (SII) has demonstrated its effectiveness as an early and convenient prognostic indicator in cases of metastatic castration resistant prostate cancer (mCRPC) and metastatic renal cell carcinoma. The study also examined its significance in predicting the outcome of stomach carcinomas. ^24^ Our study focused on examining the correlation between SII and the severity of COVID-19. However, our analysis did not find any statistically significant relationship between the two variables. Fois et al. found a strong correlation between the SII and the severity of COVID-19. Nevertheless, the study found that the area under the curve (AUC) for the SII was only marginally significant, with a p-value of 0.060. ^25^

Our study, for the first time, shows a significant association between NMR and COVID-19 severity. The AUC also showed significance at >10.34 being the cut off value for the association of NMR with severe COVID-19 disease. NMR has not been studied extensively in previous studies and may be regarded as an easy and early prognostic marker of COVID-19 severity. This is a strength of the present study.

Our study has some limitations. First, this was a single-center study with no external validation cohort. The hematological parameters studied were taken at the time of admission. It remains unclear whether stepwise changes occur when the patient’s condition worsens clinically.

Several markers, such as coagulation parameters, D-dimer, and CRP, were not included in this investigation, which might have offered a comprehensive understanding of the severity and underlying causes.

## Conclusion

Several haematological indicators have been widely investigated for predicting the severity of COVID-19. The results of our investigation revealed a noteworthy correlation between the levels of ALC (absolute lymphocyte count), NMR (neutrophil-to-lymphocyte ratio), and the severity of COVID-19. This has enhanced the significance of our investigation, as the relationship between NMR and the severity of COVID-19 has not been fully investigated. The utilisation of ALC and NMR at modest facilities and rural hospitals inside our nation, which possess limited resources, can effectively facilitate the triage and management of patients upon admission.

### Ethics and consent

The study was approved by our Institutional Review Board (IRB) known as Kasturba Medical College and Kasturba Hospital Institutional Ethics Committee (reference number: 740/2020). It was approved on 3 ^rd^ December,2020 for a period of three months till 31 ^st^ March, 2021. This study was conducted in accordance with the principles of the Declaration of Helsinki. Written informed consent was waved and telephonic verbal consent was taken citing restrictions during COVID-19. The same was approved by the Institutional Ethics Committee. The data collected were kept confidential.

## Data Availability

Figshare: Covid study dataset. DOI: https://doi.org/10.6084/m9.figshare.25299151.v1. ^26^ The data contains various hematological parameters in cases of Covid-19 of varying severity. Data are available under the terms of the Creative Commons Attribution 4.0 International license (CC-BY 4.0).

## References

[1] Zhu N Zhang D Wang W : A Novel Coronavirus from Patients with Pneumonia in China, 2019. *N. Engl. J. Med.* 2020 Feb 20;382(8):727–733. 10.1056/NEJMoa2001017 31978945 PMC7092803

[2] Andrews MA Areekal B Rajesh KR : First confirmed case of COVID-19 infection in India: A case report. *Indian J. Med. Res.* 2020 May;151(5):490–492. 10.4103/ijmr.IJMR_2131_20 32611918 PMC7530459

[3] Ministry of Health and Family Welfare, Government of India: COVID-19 India.Accessed on: 2021 April 19. Reference Source

[4] Yan Y Shin WI Pang YX : The first 75 days of novel coronavirus (SARS-CoV-2) outbreak: recent advances, prevention, and treatment. *Int. J. Environ. Res. Public Health.* 2020 Jan;17(7):2323. 10.3390/ijerph17072323 32235575 PMC7177691

[5] Friedman J Calderón-Villarreal A Bojorquez I : Excess out-of-hospital mortality and declining oxygen saturation: the sentinel role of emergency medical services data in the COVID-19 crisis in Tijuana, Mexico. *Ann. Emerg. Med.* 2020 Oct 1;76(4):413–426. 10.1016/j.annemergmed.2020.07.035 33012377 PMC7377712

[6] Looi MK : Covid-19: WHO adds JN.1 as new variant of interest. *BMJ.* 2023 Dec 21;383:383–2975. 10.1136/bmj.p2975 38128957

[7] Ministry of Health and Family Welfare, Government of India. Clinical management protocol for covid-19 (In adults); version 6. 2021 May 24;4–6. Accessed on May 30 2021. Reference Source

[8] Guan WJ Ni ZY Hu Y : Clinical Characteristics of Coronavirus Disease 2019 in China. *N. Engl. J. Med.* 2020 Apr 30;382(18):1708–1720. Epub 2020 Feb 28. 10.1056/NEJMoa2002032 32109013 PMC7092819

[9] Zhou F Yu T Du R : Clinical course and risk factors for mortality of adult inpatients with COVID-19 in Wuhan, China: a retrospective cohort study. *Lancet.* 2020 Mar 28;395(10229):1054–1062. Epub 2020 Mar 11. Erratum in: Lancet. 2020 Mar 28;395(10229):1038. Erratum in: Lancet. 2020 Mar 28;395(10229):1038. PMID: 32171076; PMCID: PMC7270627. 10.1016/S0140-6736(20)30566-3 32171076 PMC7270627

[10] Richardson S Hirsch JS Narasimhan M : Presenting Characteristics, Comorbidities, and Outcomes Among 5700 Patients Hospitalized With COVID-19 in the New York City Area. *JAMA.* 2020 May 26;323(20):2052–2059. Erratum in: JAMA. 2020 May 26;323(20):2098. 10.1001/jama.2020.6775 32320003 PMC7177629

[11] Gupta N Ish P Kumar R : Evaluation of the clinical profile, laboratory parameters and outcome of two hundred COVID-19 patients from a tertiary centre in India. *Monaldi Arch. Chest Dis.* 2020 Nov 9;90(4). 10.4081/monaldi.2020.1507 33169598

[12] Lin S Mao W Zou Q : Associations between hematological parameters and disease severity in patients with SARS-CoV-2 infection. *J. Clin. Lab. Anal.* 2021 Jan;35(1):e23604. Epub 2020 Nov 13. 10.1002/jcla.23604 33184946 PMC7843261

[13] Elshazli RM Toraih EA Elgaml A : Diagnostic and prognostic value of hematological and immunological markers in COVID-19 infection: A meta-analysis of 6320 patients. *PLoS One.* 2020 Aug 21;15(8):e0238160. 10.1371/journal.pone.0238160 32822430 PMC7446892

[14] Agrawal A Tyagi P Mahavar S : Study of hematological and biochemical parameters in a cohort of Indian COVID-19 patients admitted in a tertiary care centre. *Int. J. Adv. Med.* 2020 Dec;7(12):1840–1845. 10.18203/2349-3933.ijam20205045

[15] Hemmat N Derakhshani A Bannazadeh Baghi H : Neutrophils, crucial, or harmful immune cells involved in coronavirus infection: a bioinformatics study. *Front. Genet.* 2020 Jun 9;11:641. 10.3389/fgene.2020.00641 32582303 PMC7296827

[16] Feng X Li S Sun Q : Immune-inflammatory parameters in COVID-19 cases: a systematic review and meta-analysis. *Front. Med.* 2020 Jun 9;7:301. 10.3389/fmed.2020.00301 32582743 PMC7295898

[17] Chaplin DD : Overview of the immune response. *J. Allergy Clin. Immunol.* 2010 Feb 1;125(2):S3–S23. 10.1016/j.jaci.2009.12.980 20176265 PMC2923430

[18] Sakaguchi S Yamaguchi T Nomura T : Regulatory T cells and immune tolerance. *Cell.* 2008 May 30;133(5):775–787. 10.1016/j.cell.2008.05.009 18510923

[19] Li T Lu H Zhang W : Clinical observation and management of COVID-19 patients. *Emerg. Microbes Infect.* 2020 Dec;9(1):687–690. 10.1080/22221751.2020.1741327 32208840 PMC7103696

[20] Xu H Zhong L Deng J : High expression of ACE2 receptor of 2019-nCoV on the epithelial cells of oral mucosa. *Int. J. Oral Sci.* 2020 Feb 24;12(1):8. 10.1038/s41368-020-0074-x 32094336 PMC7039956

[21] Ziegler-Heitbrock L Ancuta P Crowe S : Nomenclature of monocytes and dendritic cells in blood. *Blood.* 2010 Oct 21;116(16):e74–e80. 10.1182/blood-2010-02-258558 20628149

[22] Gatti A Radrizzani D Viganò P : Decrease of non-classical and intermediate monocyte subsets in severe acute SARS-CoV-2 infection. *Cytometry A.* 2020 Sep;97(9):887–890. 10.1002/cyto.a.24188 32654350 PMC7404377

[23] Arango Duque G Descoteaux A : Macrophage cytokines: involvement in immunity and infectious diseases. *Front. Immunol.* 2014 Oct 7;5:491. 10.3389/fimmu.2014.00491 25339958 PMC4188125

[24] Wang Q Zhu D : The prognostic value of systemic immune-inflammation index (SII) in patients after radical operation for carcinoma of stomach in gastric cancer. *J. Gastrointest. Oncol.* 2019 Oct;10(5):965–978. 10.21037/jgo.2019.05.03 31602335 PMC6776812

[25] Fois AG Paliogiannis P Scano V : The Systemic Inflammation Index on Admission Predicts In-Hospital Mortality in COVID-19 Patients. *Molecules.* 2020 Dec 4;25(23):5725. 10.3390/molecules25235725 33291581 PMC7731255

[26] Krishnan G Karanth S Vidyasagar S : Covid study dataset.Dataset. *figshare.* 2024. 10.6084/m9.figshare.25299151.v1

