# Supplementary material for: Association between Hematological Parameters and Severity of COVID-19 disease
Source: F1000Res. 2024 Nov 8;13:517. Originally published 2024 May 20. [Version 3] doi: 10.12688/f1000research.148734.3 (PMC11472279; doi:10.12688/f1000research.148734.3)
Supplement: Peer review history

# Reviewer report: Reviewer response for version 3

**DOI:** 10.5256/f1000research.173747.r349338

**Published:** 2025-01-08

**Authors:** Tak Amit

**Affiliations:** 1 ICMR - National Centre for Disease Informatics and Research, Bengaluru, India

The article is well-written, and the methodology is correct. However, I strongly suggest that the interpretation is not sound. 

 1. The article's title focuses on the relationship between hematological parameters and COVID-19 severity. However, I suggest changing it to something like 'Relationship between various medical conditions and COVID-19 severity'. Here the history of the presence of comorbidities are good markers of COVID severity. These parameters are statistically and clinically significant here. 

 2. I emphasize that authors should consider the difference between the terms 'statistically significant' and 'clinically significant'. As they said hematological parameters are statistically significant, but ORs and AUCs showed that these parameters are not very clinically significant compared to co-morbidities like DM and HTN. 

 3. The authors took the statistically significant difference in age and gender between the two groups very casually.  As both the groups are not matched. The interpretations might be wrong. 

 4. Word severity is also very casually defined for research. Authors might consider mortality risk as they have data on survivors and non-survivors.

Is the work clearly and accurately presented and does it cite the current literature?

Yes

If applicable, is the statistical analysis and its interpretation appropriate?

Partly

Are all the source data underlying the results available to ensure full reproducibility?

Yes

Is the study design appropriate and is the work technically sound?

Yes

Are the conclusions drawn adequately supported by the results?

Partly

Are sufficient details of methods and analysis provided to allow replication by others?

Yes

Reviewer Expertise:

Electrophysiology, Public Health, Neurophysiology

I confirm that I have read this submission and believe that I have an appropriate level of expertise to confirm that it is of an acceptable scientific standard, however I have significant reservations, as outlined above.

# Reviewer report: Reviewer response for version 3

**DOI:** 10.5256/f1000research.173747.r339005

**Published:** 2024-11-11

**Authors:** Santiago J Ballaz

**Affiliations:** 1 Medical School, Universidad Espíritu Santo, Samborondón, Ecuador

The authors have taken into account all of my remarks and recommendations. The work is, in my opinion, ready for indexing. Thank you.

Is the work clearly and accurately presented and does it cite the current literature?

Yes

If applicable, is the statistical analysis and its interpretation appropriate?

Yes

Are all the source data underlying the results available to ensure full reproducibility?

Partly

Is the study design appropriate and is the work technically sound?

Yes

Are the conclusions drawn adequately supported by the results?

Yes

Are sufficient details of methods and analysis provided to allow replication by others?

Partly

Reviewer Expertise:

COVID-19 , AI, Epidemiology, Pharmacology, Neurosciences

I confirm that I have read this submission and believe that I have an appropriate level of expertise to confirm that it is of an acceptable scientific standard.

# Reviewer report: Reviewer response for version 2

**DOI:** 10.5256/f1000research.170579.r326154

**Published:** 2024-10-10

**Authors:** Santiago J Ballaz

**Affiliations:** 1 Medical School, Universidad Espíritu Santo, Samborondón, Ecuador

To the attention of Krishnan et al.,

 This work is a second version of an already revised manuscript dealing with the association of specific hematological indexes with COVID-19 severity. I was asked to take out a few subtleties that were still present in the text. I have a few important recommendations:

1. I strongly suggest that you expand on the study's limitations in the discussion section. For example: 1) the treatments taken cannot be ruled out; 2) the findings may not be applicable to future patient groups due to the rapid evolution of COVID-19 variations; 3) the study was conducted at a single center, which may restrict the findings' generalizability; etc.
2. The authors ought to provide more information about the severity criteria used in the patient classification process. Research organizations reporting hematological findings associated with the severity of COVID-19 differ greatly in these criteria. Because of this, comparing different studies becomes too challenging.
3. Regarding the SII index, please mention the following reference in the text: ^1^ It corroborates some of your findings.
4. The phrase "COVID-19 infection" is not suitable. Instead, use the term "COVID-19 disease," which is primarily caused by infection with Sars-CoV-2.

Is the work clearly and accurately presented and does it cite the current literature?

Yes

If applicable, is the statistical analysis and its interpretation appropriate?

Yes

Are all the source data underlying the results available to ensure full reproducibility?

Partly

Is the study design appropriate and is the work technically sound?

Yes

Are the conclusions drawn adequately supported by the results?

Yes

Are sufficient details of methods and analysis provided to allow replication by others?

Partly

Reviewer Expertise:

COVID-19 , AI, Epidemiology, Pharmacology, Neurosciences

I confirm that I have read this submission and believe that I have an appropriate level of expertise to confirm that it is of an acceptable scientific standard, however I have significant reservations, as outlined above.

## References

[1] : Predictive Value of the Platelet Times Neutrophil-to-Lymphocyte Ratio (SII Index) for COVID-19 In-Hospital Mortality. *EJIFCC* .2023;34(2) :167-173 37455846 PMC10349308

# Response

**Published:** 2024-10-19

**Authors:** Shubhada Karanth

**Affiliations:** General Medicine, Kasturba Medical College Manipal, Udupi, Karnataka, India

Dear Dr. Ballaz , thank you for reviewing our paper and your input. We have tried our best to make modifications according to  your comments. Please find our reply to your comments below.

 Reply 1)We have elaborated on the limitations of our study in further detail.

 Reply 2) The criteria we used for classifying our cases on the basis of criteria suggested by the Indian Council of Medical Research. We have explained it in further detail for better clarity.

 Reply 3) The mentioned reference has been added.

 Reply 4) The term “COVID-19 infection” has been replaced by “COVID-19 disease” as suggested.

# Reviewer report: Reviewer response for version 2

**DOI:** 10.5256/f1000research.170579.r319841

**Published:** 2024-09-18

**Authors:** Yogesh Kumar

**Affiliations:** 1 Physiology, All India Institute of Medical Sciences, Patna, India

Thank you for sending the revised version of the manuscript titled *"Association Between Hematological Parameters and Severity of COVID-19 Infections"*.

 I have carefully reviewed the modifications made in response to the reviewer’s comments.

 I am pleased to confirm that the authors have effectively addressed all the concerns raised in the initial review.

 The inclusion of the study by Rizo Téllez et al. and the elaboration of the introduction significantly enhance the context of the work. Additionally, the expanded discussion of JN1 and the study’s relevance to clinical practice, particularly in resource-limited settings, is both appropriate and valuable.

 Given these improvements, I am happy to approve the revised version for acceptance.

 Thank you again for your patience and efforts. 

 Regards

Is the work clearly and accurately presented and does it cite the current literature?

Partly

If applicable, is the statistical analysis and its interpretation appropriate?

Yes

Are all the source data underlying the results available to ensure full reproducibility?

Partly

Is the study design appropriate and is the work technically sound?

Yes

Are the conclusions drawn adequately supported by the results?

Yes

Are sufficient details of methods and analysis provided to allow replication by others?

Partly

Reviewer Expertise:

Neurophysiologist.

I confirm that I have read this submission and believe that I have an appropriate level of expertise to confirm that it is of an acceptable scientific standard.

# Reviewer report: Reviewer response for version 1

**DOI:** 10.5256/f1000research.163082.r291337

**Published:** 2024-07-02

**Authors:** Yogesh Kumar

**Affiliations:** 1 Physiology, All India Institute of Medical Sciences, Patna, India

- This manuscript addresses a timely and relevant issue with potential clinical impact. It provides valuable insights into the use of hematological parameters for predicting COVID-19 severity. With minor revisions to enhance the clarity and robustness of the findings, it would be suitable for publication.
- The study's novelty lies in its focus on easily accessible hematological parameters and their potential to predict COVID-19 severity, particularly in an Indian context. This is significant given the limited resources in many healthcare settings in India and other similar regions.
- The introduction could benefit from a more detailed discussion of previous studies that have explored similar parameters, providing a more robust rationale for the current study.
- The mention of the resurgence with the JN1 variant could be elaborated to explain its relevance to the study's timing and context.
- The method of calculating the Systemic Immune Inflammatory Index (SII) should be clearly defined.
- The limitations section should be expanded to discuss the impact of the single-center design and the lack of external validation in more detail.
- The discussion could benefit from a more thorough exploration of the clinical implications of the findings, particularly in the context of resource-limited settings.

Is the work clearly and accurately presented and does it cite the current literature?

Partly

If applicable, is the statistical analysis and its interpretation appropriate?

Yes

Are all the source data underlying the results available to ensure full reproducibility?

Partly

Is the study design appropriate and is the work technically sound?

Yes

Are the conclusions drawn adequately supported by the results?

Yes

Are sufficient details of methods and analysis provided to allow replication by others?

Partly

Reviewer Expertise:

Neurophysiologist.

I confirm that I have read this submission and believe that I have an appropriate level of expertise to confirm that it is of an acceptable scientific standard, however I have significant reservations, as outlined above.

## References

[1] : Role of Hematological Parameters in the Grading of COVID-19 and a Model to Predict the Outcome in Inpatients. *Cureus* .2023;15(9) : 10.7759/cureus.45276 e45276 10.7759/cureus.45276 37846240 PMC10576849

# Response

**Published:** 2024-08-19

**Authors:** Shubhada Karanth

**Affiliations:** General Medicine, Kasturba Medical College Manipal, Udupi, Karnataka, India

Dear Dr Kumar, we would like to thank you for your time and valuable input. We have tried our best to consider your points. Kindly consider reviewing them.

  

- Comment :The introduction could benefit from a more detailed discussion of previous studies that have explored similar parameters, providing a more robust rationale for the current study.
- Reply: We have included a more detailed discussion
- Comment: The method of calculating the Systemic Immune Inflammatory Index(SII) should be clearly defined
- Reply: We have added the method of calculating SII
- Comment: The mention of the resurgence with the JN1 Variant could be elaborated to explain its relevance to the study’s timing and context.
- Reply: We have elaborated on the resurgence of JN1 and its relevance as suggested.
- Comment: The limitations section should be expanded to discuss the impact of the single-center design and the lack of external validation in more detail
- Reply: We have added the points under limitations
- Comment: The discussion could benefit from a more thorough exploration of the clinical implications of the findings, particularly in the context of resource-limited settings
- Reply: We have provided a more detailed exploration of the clinical implications

# Reviewer report: Reviewer response for version 1

**DOI:** 10.5256/f1000research.163082.r285245

**Published:** 2024-06-18

**Authors:** Marc Vasse

**Affiliations:** 1 Biology Department,, Foch Hospital ., Suresnes, France

In this paper, the authors have analyzed different hematological parameters, including ratios derived from the differential, in order to identify the severity of COVID-19; It is a retrospective study performed on 573 patients, during the first wave of COVID-19. hey observe that lymphopenia and the neutrophil to monocyte ratio is predictive of the severity of the disease.

 It is a well-written paper. The main problem is that is that the study is not very original, since lymphopenia was very quickly identified as a marker of severity of COVID-19, as were the different differential ratios. In addition, the authors indicate in the discussion that this is the first time that the NMR ratio has been shown as a factor in the severity of COVID-19; This is incorrect, a first article indexed in 2021 by Rizo-Téllez et al (Microorganisms. 2020 Oct 10;8(10):1560. PMID: 33050487) has already described this parameter as predictive. Finally, the question that now arises is: are these markers identified during the first wave in 2020 still relevant?

Is the work clearly and accurately presented and does it cite the current literature?

Partly

If applicable, is the statistical analysis and its interpretation appropriate?

Yes

Are all the source data underlying the results available to ensure full reproducibility?

Yes

Is the study design appropriate and is the work technically sound?

Yes

Are the conclusions drawn adequately supported by the results?

Yes

Are sufficient details of methods and analysis provided to allow replication by others?

Yes

Reviewer Expertise:

Biologiclal markers in COVID-19

I confirm that I have read this submission and believe that I have an appropriate level of expertise to confirm that it is of an acceptable scientific standard, however I have significant reservations, as outlined above.

# Response

**Published:** 2024-08-19

**Authors:** Shubhada Karanth

**Affiliations:** General Medicine, Kasturba Medical College Manipal, Udupi, Karnataka, India

Dear Dr Vasse, thank you for your time and valuable input towards improving our paper. Kindly review the modifications made.

- After going through the article by Rizo Téllez et al according to your suggestion, we have removed our claim that our study was the first one to consider NMR ratio and have cited the study by Rizo Téllez et al.
- Regarding the relevance, we feel that though it may not be as relevant as before during the COVID wave, it may still its significance considering that COVID-19 is still prevalent with new variants emergin. In addition, we felt it was important to publish the findings from our center to contribute to the existing data and literature.
